# Barriers to the use of trained interpreters in consultations with refugees in four resettlement countries: a qualitative analysis using normalisation process theory

**DOI:** 10.1186/s12875-020-01314-7

**Published:** 2020-12-05

**Authors:** Anne MacFarlane, Susann Huschke, Kevin Pottie, Fern R. Hauck, Kim Griswold, Mark F. Harris

**Affiliations:** 1 School of Medicine and Health Research Institute, University of Limerick, Limerick, Ireland; 2 Bruyère Research Institute, Department of Family Medicine, University of Ottawa, Ottawa, Canada; 3 Department of Family Medicine, University of Virginia, Charlottesville, USA; 4 Department of Family Medicine, University at Buffalo, Buffalo, USA; 5 Centre for Primary Health Care and Equity, University of New South Wales, Sydney, Australia

**Keywords:** Refugees, Communication, Primary health care, Health equity, Health resources, Surveys and questionnaires

## Abstract

**Background:**

Increasing numbers of primary care practitioners in refugee resettlement countries are providing care to refugees. Access to trained interpreters is a priority for these practitioners, but there are many barriers to the implementation of interpreted consultations in routine care. There is a lack of international, theoretically informed research. The purpose of this paper is to understand barriers to interpreter use in primary care consultations in four resettlement countries using Normalisation Process Theory.

**Method:**

We conducted a cross-sectional online survey with networks of primary care practitioners (PCPs) who care for refugees in Australia, Canada, Ireland and the US (*n* = 314). We analysed qualitative data from the survey about barriers to interpreter use (*n* = 178). We completed an inductive thematic analysis, iteratively developed a Normalisation Process Theory (NPT)-informed coding frame and then mapped the emergent findings onto the theory’s construct about *enacting* interpreted consultations.

**Results:**

In all four countries, the use of an interpreter presented communication and interaction challenges between providers and patients, which can impede the goals of primary care consultations. Primary care practitioners did not always have confidence in interpreted consultations and described poor professional practice by some interpreters. There was variation across countries, and inconsistency within countries, in the availability of trained interpreters and funding sources.

**Conclusion:**

There are shared and differential barriers to implementation of interpreted consultations in a consistent and sustained way in the four countries studied. These findings can be used to inform country-specific and international level policies and interventions focusing on improving skills and resources for interpreted consultations to improve implementation of interpreted primary care consultations.

**Supplementary Information:**

The online version contains supplementary material available at 10.1186/s12875-020-01314-7.

## Background

The forced movement of people as refugees around the globe is increasing, and reached record highs in 2017 [1]. Refugee resettlement has therefore affected increasing numbers of primary care practitioners. A refugee is a person who ‘owing to a well-founded fear of being persecuted for reasons of race, religion, nationality, membership of a particular social group or political opinion, is outside the country of his nationality and is unable or owing to such fear unwilling to avail himself of the protection of that country’ [2]. Refugee health has received considerable attention from stakeholders in academic, community and health sector settings who are concerned with health equity in resettlement countries in the developed world [3]. It is clear that refugees’ access to primary healthcare is problematic: there are documented and inter-related problems stemming from variation in healthcare entitlements, and challenges navigating service that are unfamiliar and that may be poorly co-ordinated. There is also a lack of community resources available to support refugees and migrants to manage these problems.

[4–6]. One other majorreason for such problems with access relates to communication challenges in consultations due to cultural and linguistic diversity [4–7]. Informal responses to these challenges, such as the ad hoc use of family members and friends as interpreters, are common [8]. While pragmatic, this may lead directly and indirectly to inequitable health outcomes [9, 10]. The gold standard recommended in the literature is to use trained interpreters who provide culturally aware, comprehensive and safe communication [11, 12].

Healthcare systems need to establish formalised responses that promote the routine use of trained interpreters [8, 13]. Implementing interpreted consultations in primary care settings is, however, extremely complex, due to a combination of macro-, meso- and micro-level factors [14, 15] (see Table 1).

**Table 1:** Examples of macro-, meso- and micro-level influences on implementation of consultations with trained interpreters

Macro-level	Meso-level	Micro-level
Resources for interpreting services are essential but are not always available [16]	Interpreters may be inadequately trained and accredited; primary care providers may be inadequately trained to work with interpreters [8, 17]	The dynamics of interpreted consultations are unfamiliar and can feel demanding. They can present demands for establishing trusting relationships between the doctor and patient [18, 19]

Primary care practitioners working with refugees have indicated that the use of trained interpreters is a major priority for them [5, 6]. Therefore, there is a strong rationale to intensify research and development projects to investigate and support the implementation of trained interpreters in primary care [7]. It is particularly important to know more about barriers to the routine use of trained interpreters in order to design policies and interventions to overcome these. International comparative studies in resettlement countries are useful in this regard [3, 6]. A recent European study showed variation across settings in the availability and use of trained interpreters and how this presents barriers to their routine use [15, 16]. In Ireland, for example, there is a lack of trained interpreters available for use, while in the Netherlands, a policy change withdrew payments for the use of available trained interpreters [15, 16]. There has been no comparison of international settings outside Europe.

Theoretically informed studies are also important to provide a robust evidence base to support implementation initiatives [20]. This is because they provide an opportunity for accumulating generalisable knowledge. However, these are rare in the field of interpreter studies in primary care where the majority of studies are atheoretical and descriptive.

The purpose of our paper is to advance the field by analysing barriers to interpreter use in four resettlement countries using May and Finch’s [21] Normalisation Process Theory (NPT: see Table 2 and Methods section for a detailed description). An online survey with 314 primary care practitioners assessed how interpretation is used, funded and governed in primary care practices serving refugees in Australia, Canada, Ireland and the US. A key quantitative finding was that there was wide variation in interpreter use between jurisdictions (highest in the US and lowest in Ireland). These results appear to be in part due to differences in local policy and practice but have not been analysed in depth.

**Table 2:** Normalisation Process Theory (May and Finch, 2009): Description of implementation work and specific questions for implementing interpreted consultations

Description of implementation work	Key questions for implementing interpreted consultations
Sense-making	Can stakeholders see the value and potential impact of interpreting?
Enrolment	Can they organise all the relevant stakeholders to get involved in driving the implementation forward?
**Enactment**	Have they the **resources** and **skills** to introduce and use interpreting in day-to-day practice? Do they have **confidence and trust** in the interpreted consultation and does the use of interpreters **help the interaction** between doctor and patient to achieve the goals of the consultation?
Appraisal	Can stakeholders evaluate the impact of interpreters and specify ways to reconfigure practice to sustain their use as a routine way of working?

## Method

We are a team of primary care clinicians and social scientists with an interest in health equity and refugee health issues. As mentioned above, we conducted an online survey to generate quantitative and qualitative data about the use of formal interpreting agencies by primary care practitioners who work with refugees in four countries: Australia, Canada, Ireland and US.. We aimed to survey practitioners who had some involvement or an interest in refugee health. The approach differed in each country. In Australia, US and Canada an invitation email was sent to specialist networks of primary care providers (the Society of Refugee Healthcare Providers, North America, and Refugee Health Networks in Australia and Canada). In Ireland, in the absence of such networks, the email was sent to all family practitioners (Irish College of General Practitioners members) with a request asking all those with an interest or involvement in refugee health to respond. The email was also sent to an international general primary care network (North American Primary Care Research Group). Those who responded to the open qualitative questions and who discussed their experiences of working with trained interpreters presented an information rich sample [22] for the purpose of this qualitative analysis.

We developed and piloted a questionnaire about practice characteristics (languages spoken by respondent/practice staff, access to professional interpreters); patient characteristics (languages spoken, country of origin); and interpreter use (proportion of patients for whom interpreters are used, barriers and impact of using interpreters, cost and how funded). There were also open text questions, which are the focus of the analysis for this paper (see Table 3). We administered the questionnaire using a secure online survey (Qualtrics 2018, Provo, UT).

**Table 3:** Open text questions about interpreter use, responses and emergent themes

Open-ended question	Number of free text responses/total number of responses	Emergent themes*
What negative impact have you observed?	*n* = 153 (of 187) 81% Canada *n* = 24 (of 28) USA *n* = 35 (of 40) Ireland *n* = 66 (of 80) Australia *n* = 25 (of 33) Other *n* = 3 (of 6)	(1). Inhibits the relationship between patient and provider (2). Too time-consuming (3). Interpreters not translating directly/properly (4). Interpreters overstepping their role (5). Concerns about breach of confidentiality (6). Background of interpreter not suitable (7). Costs (8). Technical problems (9). Patients refuse interpreter
Please write any other information you would like to provide.†	Total number of comments/responses to this question: *n* = 89 (of 187) Number of responses by country: Canada *n* = 11 (of 28) USA *n* = 15 (of 40) Ireland *n* = 41 (of 80) Australia *n* = 18 (of 33) Other *n* = 4 (of 6	(1). No access to formal interpreter services (2). Recommendations for changes in policy and practice (3). Comments/feedback on the survey (4). Problems with implementing interpreter services (5). Positive impact of using interpreter services

* The themes are numbered according to the number of responses that were coded for that theme, starting with the themes with the highest density

† Some responses to this question raised issues regarding the negative impact of using interpreter services and were coded into the themes that emerged from question 1

Our research had ethical approval from committees in the authors’ jurisdictions.

For this paper, we focused on anonymised responses to the open text questions and conducted an inductive analysis following the principles of framework analysis [23]. Some responses were clearly not about the use of formal interpreting agencies, referring for example to the use of a family member as interpreter. These data were not included in the analysis because, as mentioned above, our interest was in data from ‘information rich’ respondents [22]: GPs with experience of working with refugees and accessing formal interpreters.

There were nine emergent themes relating to barriers about interpreter use (see Table 3).

We then mapped the emergent themes onto NPT (see [24] for a detailed description of the mapping process). The rationale for using NPT is that it is a contemporary sociological theory designed to provide a heuristic device to ‘think through’ the implementation of innovations and interventions in healthcare settings [21]. It has four interconnected constructs that relate to each other in a fluid, rather than linear, way in real ‘space and time’. Each construct in NPT focuses on a particular kind of implementation work that stakeholders need to engage in to introduce a new way of working with a view to embedding it into their daily practice to the point that it becomes a routine, *normalised* way of working. NPT has been previously used successfully in international research about implementing interpreters in primary care [15, 25] and we saw an opportunity to add to theoretically informed literature in this field. The emergent themes in this analysis are related to the NPT construct about enactment. This is a function of the questions asked in the survey, which were about actual use of interpreters. The focus of this construct is on skills and resources for a new way of working, whether there is confidence in it and whether it helps interactions to achieve the goals of consultations (see Table 2).

To enhance quality and rigour of the analysis, the first and second authors iteratively developed an NPT coding frame (see Supplementary File 1), discussing how NPT related to the emergent themes and comparing our coding [23]. We explored whether data fell outside the focus of NPT; there were none that did.

## Results

The survey response rates varied by country and were within average ranges for external surveys (10–15%) in all countries except Ireland: Australia 26%, Canada and US (networks overlapped) 12.3%, and Ireland 5.9%. The response rate for free text questions is provided in Table 3.

Most respondents included in this qualitative analysis were over 40 years of age (*n* = 122, 68.5%), female (*n* = 131, 73.6%) and were not a refugee or an immigrant (*n* = 139, 78%).

The majority were physicians/doctors (*n* = 146, 82%), followed by nurses (*n* = 14, 7.9%) nurse practitioners or physician assistants (*n* = 8, 4.5%), allied health practitioners (*n* = 5, 2.8%) and another professional role (*n* = 5, 2.8%). Almost two-thirds were working in a practice using formal interpreters (*n* = 11; 62.5%).

Findings are presented based on our NPT analysis, starting with the most dominant themes (i.e., themes with the highest density of data).

### Interaction problems workability: interference with the goals of the consultation

In all four countries, respondents referred to the negative impact of working with interpreters on the goals of the consultation. They felt that the interpreter’s presence impedes the interaction and communication between clinician and patient. Rather than improving the consultation, respondents felt that sometimes rapport and relationship development between doctor and patient can be disrupted by the presence of an interpreter as a third party. Respondents expressed that ‘*nuances are easily missed*’ (Irish respondent 14), that it is ‘*hard to make meaningful social connections through an interpreter (relationship becomes very formal)*’ (US respondent 37), and that the ‘*patient [is] sometimes uncomfortable discussing medical complaints with a stranger*’ (Irish respondent 59).

Some of the issues appear to be related to the use of technology (i.e. telephone interpreting): it can be ‘*difficult to build rapport as refugees tend to look at the phone as they speak*’ (Australian respondent 15). Particularly in relation to more complex issues, respondents felt that they were unable to achieve the goals of the consultation: ‘*I have wondered if answers regarding mental health issues or sensitive issues regarding sexuality might be different if able to ask without an interpreter*’ (US respondent 17).

Respondents also highlighted that finding a suitable interpreter and then including them in the consultation slows them down significantly, and thereby disrupts the goal of 10–15-min consultations: it ‘*takes a lot of extra time which is generally not built into the schedule nor compensated*’ (US respondent 33). In some cases, this leads doctors to not use formal interpreter services and rely on informal interpreting or machine translation (e.g. Google Translate) instead: ‘*I have not used translation services recently as found them difficult to use in the context of a busy surgery. Most of our Somalian/African patients […]* *bring a member of the family with them*’ (Irish respondent 37).

*Relational problems: Lack of confidence and trust.*

Respondents in all four countries report a lack confidence in many aspects of interpreters’ practice, for example incomplete or elaborated interpretation and adjusted information transfer based on moral judgements, e.g. around contraception use:

*I always worry the interpreter shortens the answer or changes the question to get a yes or no answer from the patient.* (Irish respondent 6)

*It is obvious that when we are talking about sensitive information particularly mental health issues, the interpreters can be uncomfortable with the subject matter and at times appear to be either leading the patient/family member to an answer or providing a negative answer for them.* (US respondent 17).

*Potentially giving patient medical advice that is different than what I have said. (Canadian respondent 12).*

*Interpreter being unprofessional – asserting own opinion, telling client how to answer questions,* etc. (Australian respondent 11).

More specifically, some respondents suspected that the cultural/ethnic background and the gender of the interpreter occasionally impinge on the quality of the interpretation:

*With specific male interpreters (particularly when interpreting for females) I suspect they are not fully interpreting what I say or asking sensitive questions*. (US respondent 3).

*There are issues with variability in the quality of interpretation and negative attitudes of interpreters towards patients (*e.g. *some Kurmanji speaking interpreters seem to have biases against Kurmanji speaking Yazidi patients).* (Canadian respondent 25).

Furthermore, many expressed a concern about breaches in confidentiality, particularly when working with small communities where the interpreter and patient know each other, and when the consultation referred to stigmatised topics such as HIV or mental health issues.

### Contextual problems: lack of resources

Respondents identified two key issues that relate to the lack of resources needed to work with formal interpreters: prohibitive costs and/or the unavailability of formal interpreters. A number of them stressed that the costs of using formal interpreters constitute an important barrier, particularly for smaller primary care clinics:

*I wish there were interpreters at the office I work at, but the cost of an interpreter per visit is more than how much we get paid per visit.* (US respondent 5).

*There is no funding available for interpreters in primary care in Ireland. We have to pay for interpreters from the practice. We often spend more on an interpreter than we get paid for the patient for the year. As a result we cannot get interpreters for each appointment.* (Irish respondent 55).

*In Calgary, we no longer have access to in-person interpreters because Alberta Health Services has discontinued this service and Calgary Immigrant Services no longer has a volunteer program but charges patients for the cost of the interpreter which is prohibitive.* (Canadian respondent 25).

*No formal interpreters available in the area where I work. At times, communication can be very difficult, relying on under aged relatives for consultations. […] Asylum seekers have no access to interpreters.* (Irish respondent 24).

In Australia, where formal interpreters are widely integrated into the health system, respondents pointed out that there are nevertheless issues with access:

*No interpreters available for new language groups coming to Australia. If Australia accepts family with a new language there must be considerable investment at the national level to enable safe communication with the family!* (Australian respondent 8).

*Some days it is difficult to get certain interpreters (*e.g. *female Somali interpreters on Fridays). And there is a national shortage of Kunama interpreters – it would be great to have a couple more.* (Australian respondent 22).

### Skill set problems: insufficient skills

Some respondents highlighted the need for better training and a standardised accreditation process to improve the skills of specialised medical interpreters:

*There are good interpreters and less good interpreters. The good ones are terrific. The less good ones could be terrific with more training*. (US respondent 15).

*Sometimes in person interpreters are not fully trained in health, so take short cuts in translating.* (Canadian respondent 14).

A small number of respondents also stressed that physicians may similarly lack important skills and suggested that ‘*more training of how to work with interpreters and patients for medical students and trainee doctors*’ is needed (Australian respondent 20).

## Discussion

### Summary of key findings

In all four countries studied, the use of an interpreter can present challenges to doctor-patient communication and interactions, which can impede the goals of primary care consultations. Feelings of confidence and safety can also be compromised for primary care practitioners, and they described poor professional practice by some interpreters. This leads to mistrust in the work of interpreters. There is variation across countries and inconsistency within countries in the availability of trained interpreters and funding sources, highlighting contextual barriers that impact negatively on the implementation of interpreted consultations.

### Methodological critique

The analysis in this paper is based on responses to open questions in a survey, which was distributed via email to general and specialist networks of primary care providers in resettlement countries in western democracies. Determining the denominator is challenging but, as previously mentioned, the response rates fell within average rages for external surveys (10–15%) in all countries except Ireland. This may be because the survey in Ireland was administered to a general network. This means that recipients would not necessarily have experience of working with refugees and may not have seen the relevance of participating in the survey. For a qualitative analysis such as the one reported here, however, the key issue is not the representativeness of the sample but whether data included are from respondents who are information rich, meaning that they have relevant experiences to provide an account of the phenomenon of interest, i.e. they have experience of using interpreters from formal agencies in consultations with refugees. All participants included in this analysis meet this criterion.

The data are based on free text responses and are not as in-depth as data from a qualitative study using methods such as interviews or focus groups. The analysis, however, was thorough, with the use of inductive and deductive coding and a high inter-rater reliability to enhance quality and rigour. The findings resonate with existing literature [5, 6, 15], indicating their veracity and trustworthiness.

A key strength of the study design is that it brings a *theoretical* lens to the *comparative* analysis of data about barriers from *international* settings. The use of theory to inform data generation and analysis is rare in the field of migrant health and in studies about interpreting. Our critical use of NPT for theoretical analysis is, therefore, noteworthy and provides the basis for accumulating knowledge about implementation of interpreted consultations. The comparative analysis in four resettlement countries provides empirical evidence for country-specific and international actions.

### Connections with the literature

Three of the four elements presented about the NPT construct about enactment relate strongly to the need for training and skills for interpreters and primary care practitioners), which is in keeping with previous research, e.g. [6, 8]. Notwithstanding the challenges of building trust in interpreting services, particularly in small communities [18, 26, 27], the negative interactional effects in the consultation and the issue of confidentiality and trust could be remedied if interpreters were fully trained, accredited and monitored with an appropriate quality standard [17]. Awareness-raising campaigns among primary care practitioners and training for them to work with interpreters could ensure that they (i) understand the importance of using trained interpreters and (ii) have skills to feel comfortable and confident in interpreted consultations. Involving migrants and community interpreters in the training of primary care practitioners for interpreting is impactful [15, 28] and should be considered in postgraduate and continuing medical education courses.

The other key issue is about contextual barriers. Primary care providers have limited time in each encounter, often related to remuneration. Longer consultations can be difficult to schedule, as can arranging an in-person interpreter. It is important that primary care services are resourced by local, regional and/or national health authorities in order to enable primary care practitioners to use trained interpreters as a normalised practice [16]. It is worth comparing the two countries with the most obvious differences in practice: Ireland and Australia. In Ireland, there simply are no interpreter services in some areas and previous research shows that few interpreters in Ireland have training [17]. Thus, the implementation journey is at the very beginning in terms of *establishing* a funding source and supply of trained interpreters. In Australia, where interpreter services are established, the implementation issues are further on in terms of *expanding* and *improving* accessibility and use: reducing inappropriate role behaviour by interpreters, needing specific dialects, or availability on weekends. In all settings, the availability *of sustained* funding is important. Decisions about resources for interpreting can be shaped by broader political changes leading to cuts in services when anti-immigration sentiments rise [16].

### Implications for policy and practice

This theoretically informed comparative analysis of data across international settings makes an important contribution to the literature because it reveals shared and differential barriers to the use of trained interpreters in the four countries studied. It also revealsinconsistency in service provision for refugees within and across countries. This is particularly problematic given that refugees have higher incidence of mental health conditions than the general population, often related to trauma [5]. Consultations with them require effective communication and highly trained interpretation. This inconsistency would not be acceptable for implementation of other guidelines or interventions in primary care (e.g. implementation of diabetes care) and points to health inequities for refugee health. In line with WHO recommendations, inter-sectoral activity by health researchers and providers is important to emphasise health equity and the development of evidence-based policy to ensure that primary care is accessible to refugees and other migrants [13, 29].

These findings can be used to inform policies and interventions within countries that are specific to the barriers experienced in each setting. In Ireland for example, the focus could be on establishing a community of trained interpreters while in Australia it could be on innovation to extend service provision. Findings can also inform international agencies and professional bodies who provide leadership to national governments. They could reiterate that trained interpreters is the gold standard recommended in the literature and that it is a necessary feature of health services to meet health equity goals. Taken together, such national and international initiatives can minimize barriers to the use of trained interpreters and optimize communication between refugee and migrants and their primary care providers.

## Conclusions

There are shared and different barriers to implementation of interpreted consultations across countries in this survey. These reflect where each country is in terms of their implementation of trained interpreters in routine practice. There is an important opportunity to learn from experiences across countries, especially to find solutions to common barriers. Country-specific and international level policies and interventions focusing on improving skills and resources for interpreted consultations need to be developed to improve the uptake and workability of interpreting in primary care consultations.

## Supplementary Information

**Additional file 1.** :

## Data Availability

The datasets used and/or analysed during the current study are available from the corresponding author on reasonable request.

## Abbreviations

NPT: Normalisation Process Theory
US: United States
