# Supplementary material for: Barriers to the use of trained interpreters in consultations with refugees in four resettlement countries: a qualitative analysis using normalisation process theory
Source: BMC Fam Pract. 2020 Dec 5;21:259. doi: 10.1186/s12875-020-01314-7 (PMC7719256; doi:10.1186/s12875-020-01314-7)
Supplement: Supplementary file 1 — Additional file 1. : [file 12875_2020_1314_MOESM1_ESM.docx]

**Title:** Barriers to the use of trained interpreters in consultations with refugees in four resettlement countries: A qualitative analysis using Normalisation Process Theory

**Supplementary File 1: NPT Coding Frame**

| **NPT construct Enactment** | **Data sources** |
| --- | --- |
| **Contextual integration i.e.**  Focus on organisational-level supports | Resource available, or not, to cover costs of interpreted consultation  Knowledge of policy/legal context |
| **Skill set workability i.e.**  Focus on training and competency to participate in an interpreted consultation | The training/lack of training of interpreters, doctors to work in interpreted consultations  Knowledge and awareness about interpreter training |
| **Relational integration i.e.**  Focus on issues of quality, confidence and risk as overarching issues | The trust and confidence that doctors have in the interpreted consultation  Does it feel safe? Accurate? Of good quality?  Is the interpreted consultation safe for patients? Will confidentiality be observed? Are there any ethnic tensions between the interpreter and patient? |
| **Interactional workability i.e.**  Focus on the face-to-face consultation as it happens | The use of an interpreter and its impact on the face-to-face encounter, how does it feel to be in a three-way consultation?  The way the presence of the interpreter impacts on the process and the goals of the consultation, e.g. trusted communication; short consultations  Is the interpreter/doctor clear about their particular role? Do they overstep their role? |
